# Supplementary material for: Comprehensive mapping of SARS-CoV-2 interactions in vivo reveals functional virus-host interactions
Source: Nat Commun. 2021 Aug 25;12:5113. doi: 10.1038/s41467-021-25357-1 (PMC8387478; doi:10.1038/s41467-021-25357-1)
Supplement: Supplementary file 1 — Supplementary Information [file 41467_2021_25357_MOESM1_ESM.pdf]

Supplementary Information

Comprehensive mapping of SARS-CoV-2 interactions *in vivo* reveals functional virus-host interactions

Siwy Ling Yang, Louis DeFalco, Danielle E. Anderson, Yu Zhang, Ashley J Aw, Su Ying Lim, Xin Ni Lim, Kiat Yee Tan, Tong Zhang, Tanu Chawla, Yan Su, Alexander Lezhava, Andres Merits, Lin-Fa Wang, Roland G. Huber, Yue Wan

Supplementary Figure 1 to 15

Supplementary Table 1

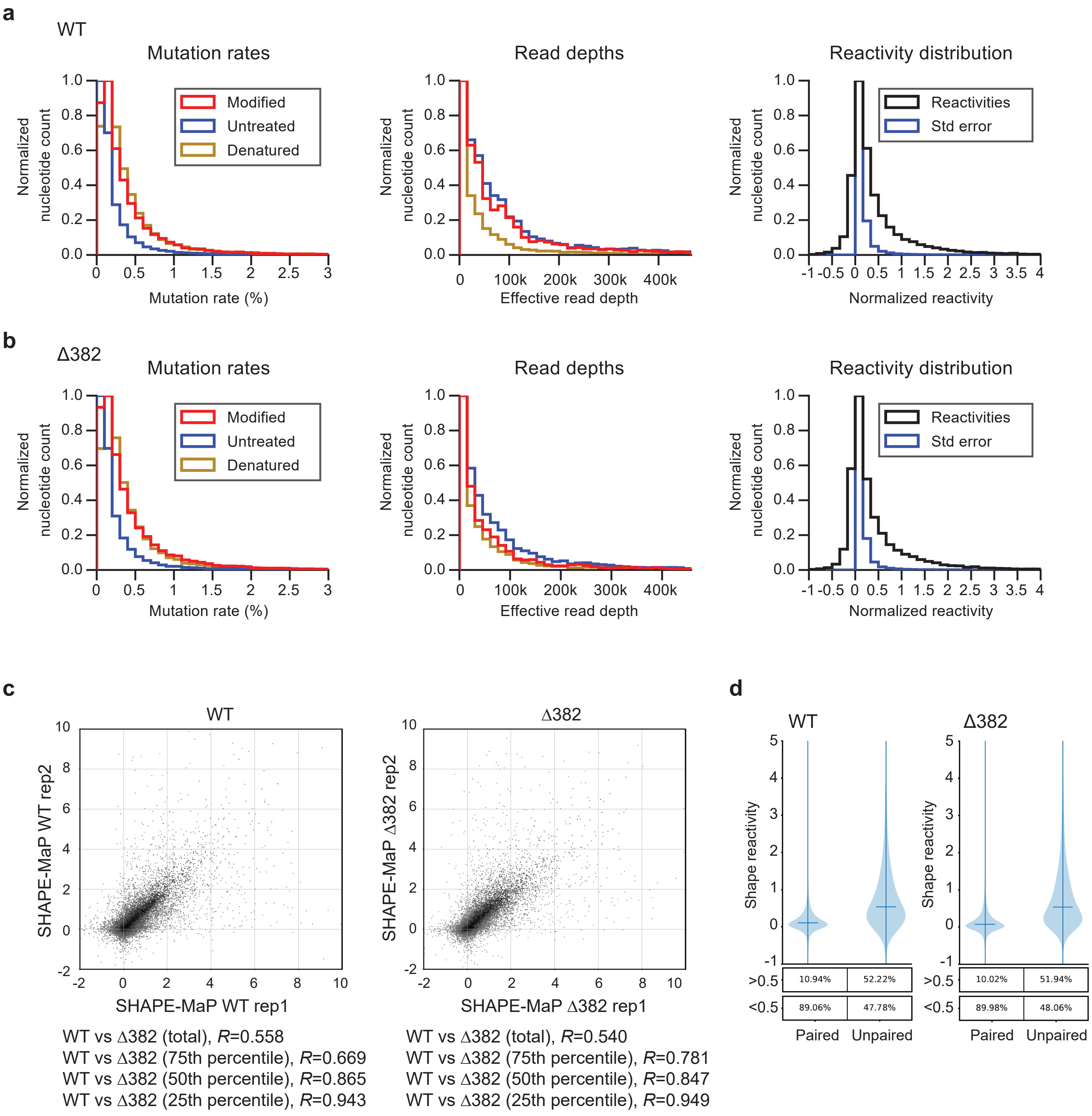

Supp. Figure 1. Quality matrixes for SHAPE-MaP for SARS-CoV-2. a,b, Mutation rates (left) and read depth (middle) for modified, untreated and denatured WT (**a**) and Δ382 (**b**) SARS-CoV-2. Distribution of the SHAPE-MaP reactivities and standard error between replicates is shown on the right. **c** Scatterplot of the SHAPE-MaP reactivity for 2 biological replicates of WT (left) and Δ382 (right). **d** Violin plots showing the SHAPE-MaP reactivities in WT (left) and Δ382 SARS-CoV-2 for paired and unpaired bases. Source data are provided as a Source Data file.

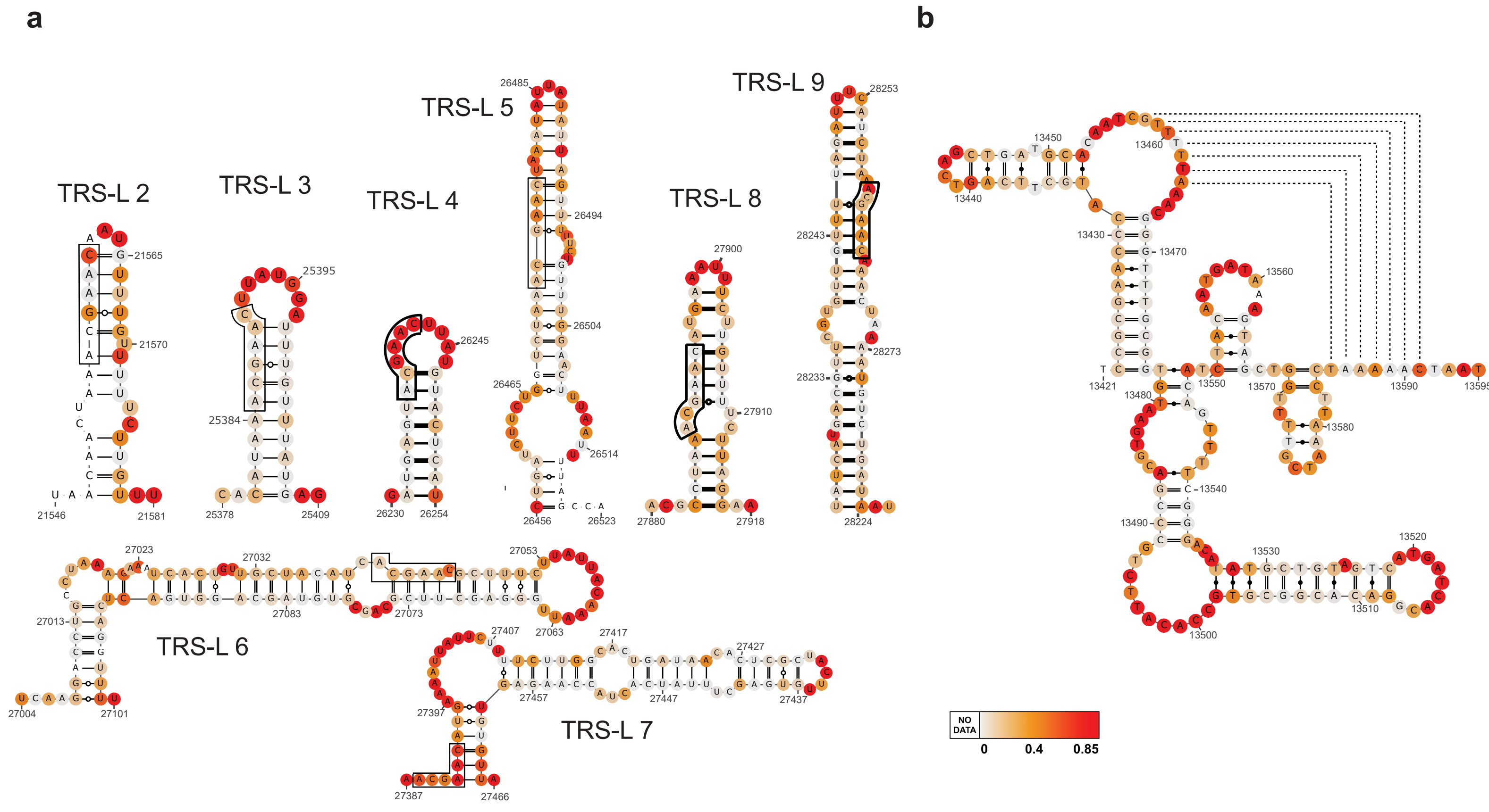

Supp. Figure 2. Structure modelling of SARS-CoV-2 genome. a,b Structure models are generated using the program RNAstructure, using SHAPE-MaP reactivities as constraints for the TRS-L elements along the genome (**a**) and for the frameshifting element using the pseudoknot function of RNAstructure (**b**). The SHAPE-reactivities are mapped onto the structure models.

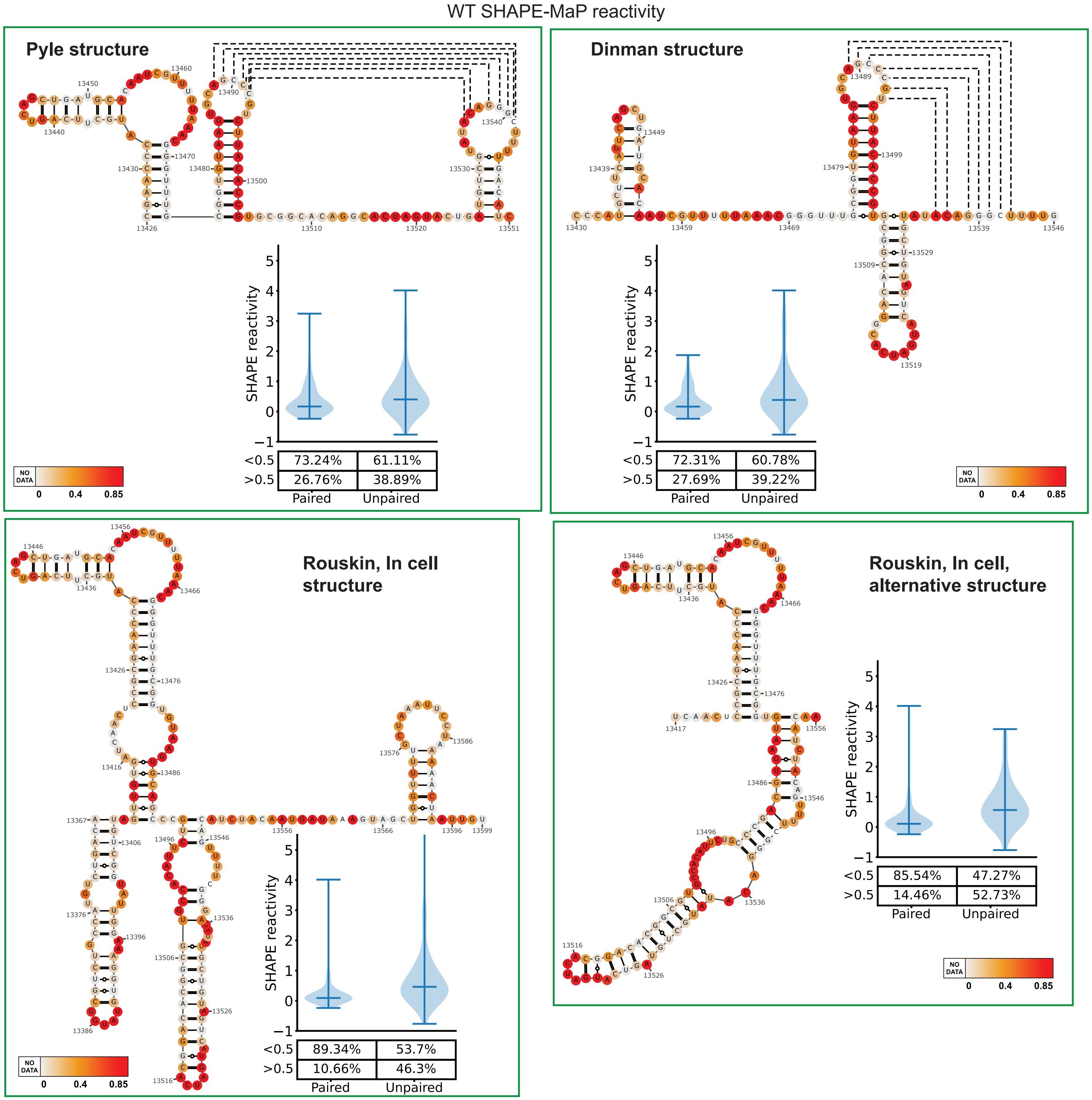

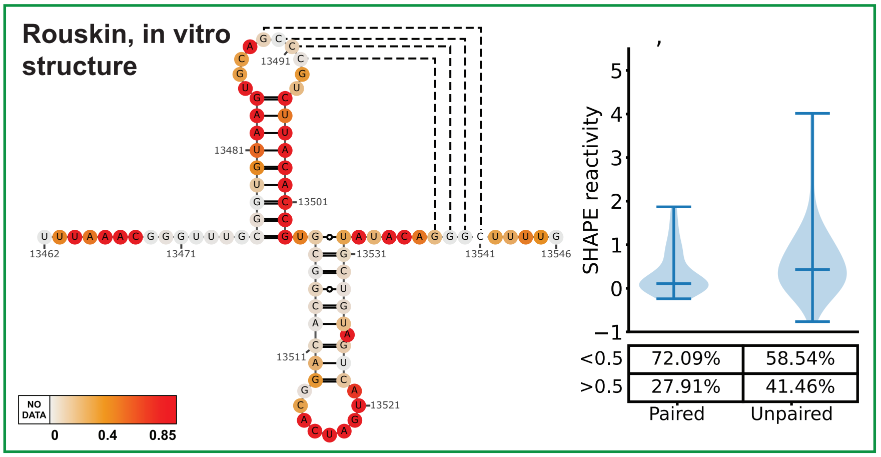

Supp. Figure 3. Mapping of WT SARS-CoV-2 SHAPE-MaP reactivities onto proposed structure models of the frameshifting element. SHAPE-MaP reactivities are mapped to 5 proposed models for the frameshifting element in SARS-CoV-2. For each structure, we calculated the percentage of bases that match double or single-stranded bases in the structure. We observed the closest concordance between our SHAPE-MaP reactivity and Lan et al (Rouskin) in cell structure model. Source data are provided as a Source Data file.

a

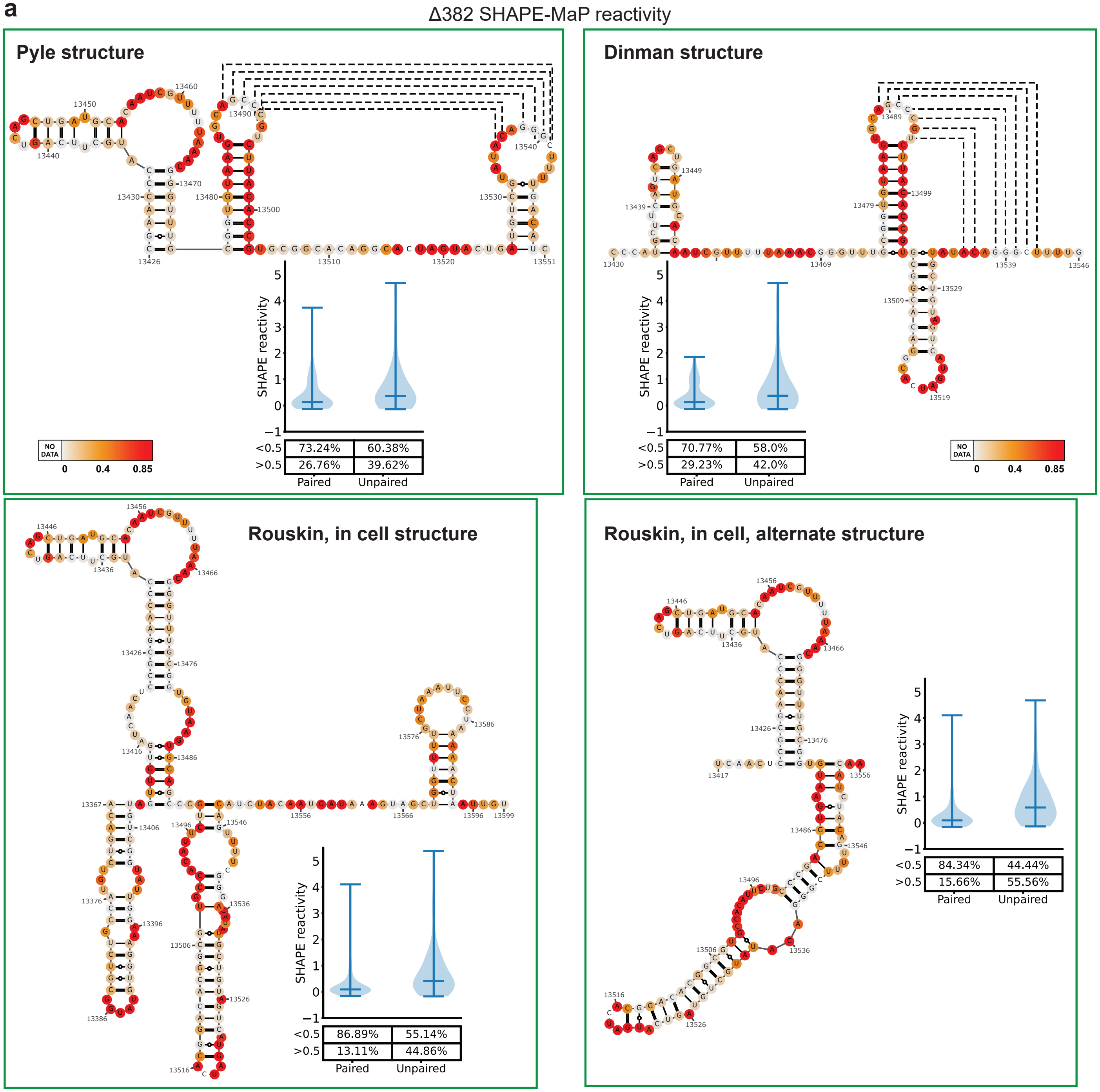

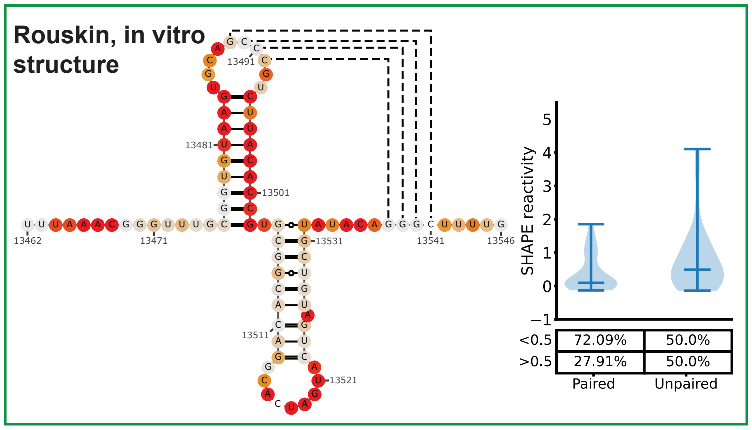

b

	WT	Delta382	DEN1	ZILM
Base Count	29844	29463	10735	10807
Paired Bases	17060	16968	5396	5976
Paired Bases (%)	57.16	57.59	50.27	55.3
Median Base Pair Span (nt)	26	26	35	32
Average Base Pair Span (nt)	60.76	64.05	84.4	75.05
Median Helix Length (nt)	5	5	4	5
Average Helix Length (nt)	5.54	5.54	5.06	5.48
Max Helix Length (nt)	27	28	24	27
Min Helix Length (nt)	1	1	1	1

Supp. Figure 4. Mapping of Δ382 SARS-CoV-2 SHAPE-MaP reactivities onto proposed structure models of the frameshifting element. a SHAPE-MaP reactivities are mapped to 5 proposed models for the frameshifting element in SARS-CoV-2. For each structure, we calculated the percentage of bases that match double or single-stranded bases in the structure. We observed the closest concordance between our SHAPE-MaP reactivity and Lan et al (Rouskin) in cell structure model. **b** Table of base-pairing information in modelled RNA structures from WT, Δ382 SARS-CoV-2, DENV and ZIKV. Source data are provided as a Source Data file.

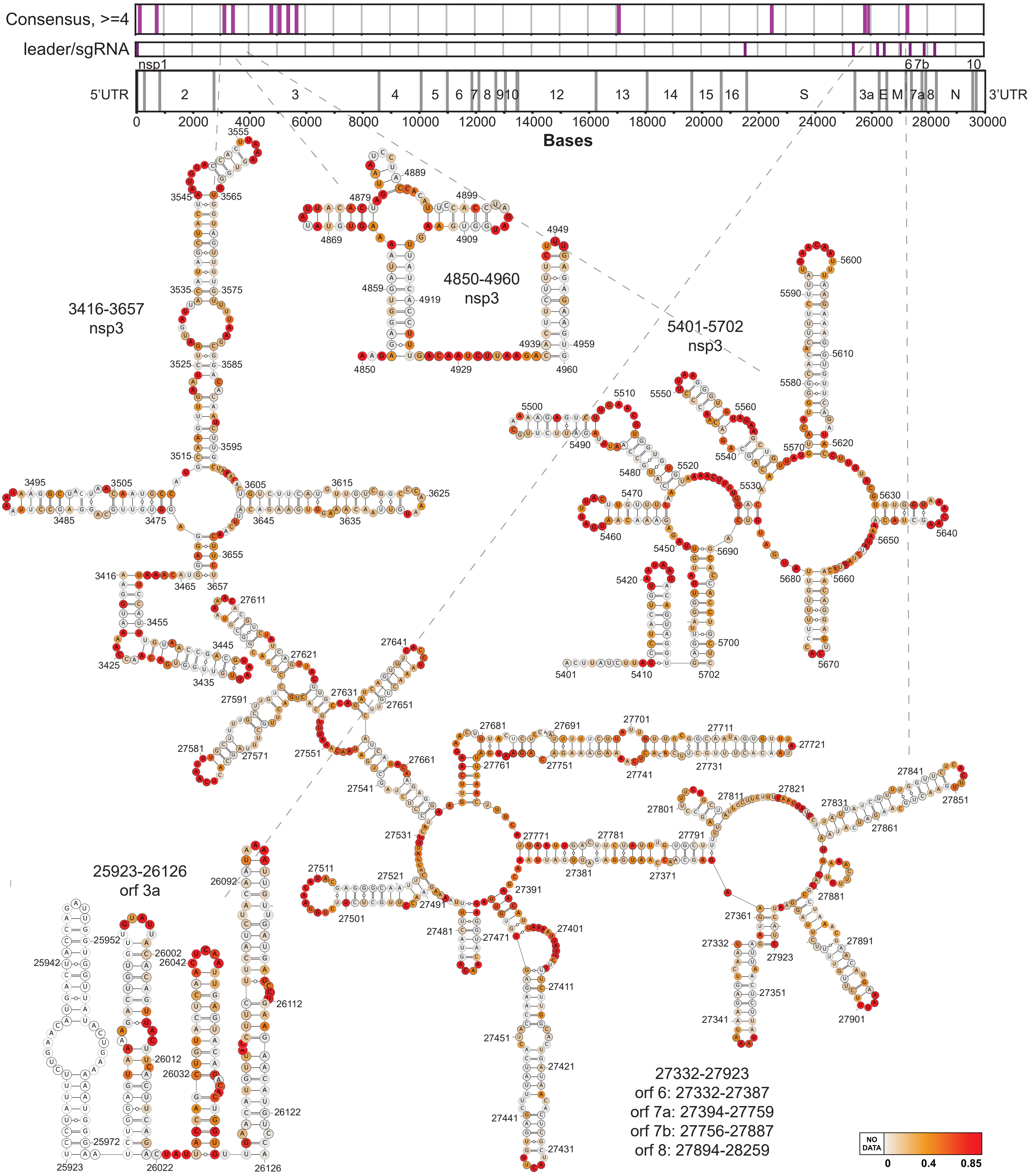

Supp. Figure 5. Functional structural elements along the SARS-CoV-2 genome. a 12 consensus regions are consistently highlighted (4/6) across both WT and Δ382 genomes and are shown in purple. **b** Structure models of 5 consensus were generated using the program RNAstructure, using SHAPE-MaP reactivities as constraints, and visualized using VARNA. The SHAPE-reactivities are mapped onto the structure models. Source data are provided as a Source Data file.

a

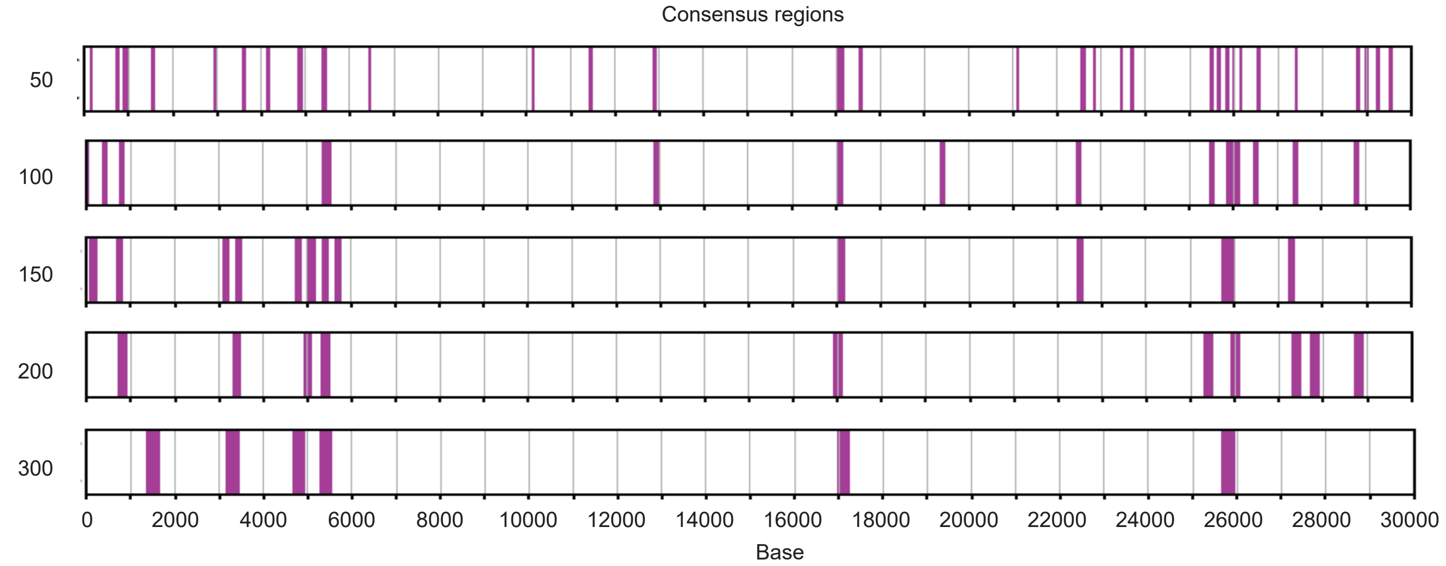

b

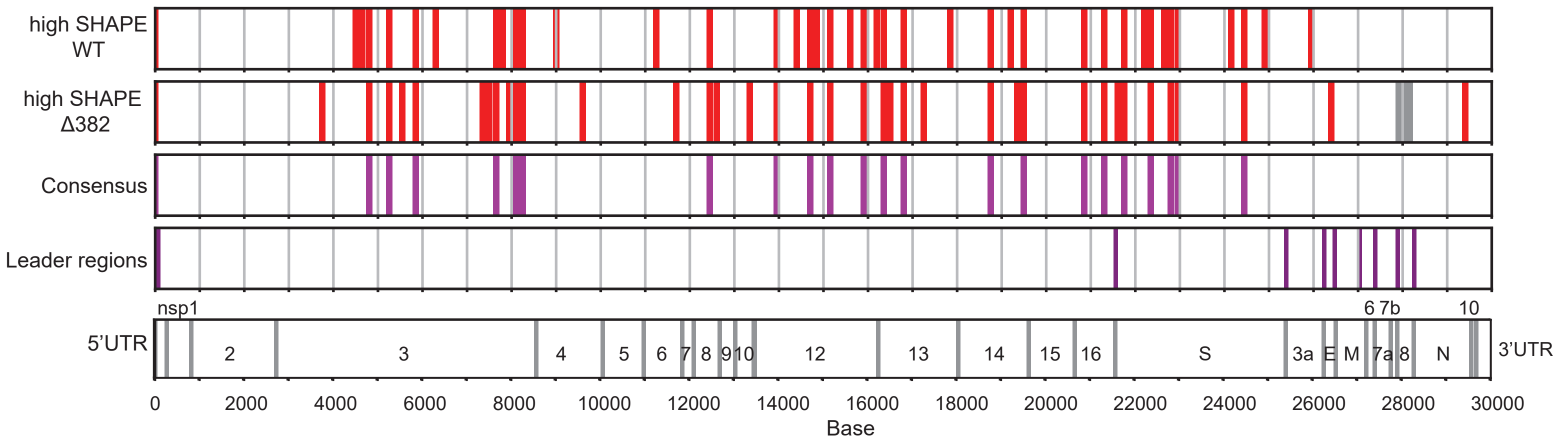

Supp. Figure 6. Highly structured and accessible regions along the SARS-CoV-2 genome. a Consensus regions (4/6 regions) along the SARS-CoV-2 identified by using different window sizes from 50 to 300 bases. **b** Highly accessible (top 20% high SHAPE-reactivity regions) regions identified along WT and Δ382 SARS-CoV-2 genome (red). Regions that show high accessibility in both WT and Δ382 SARS-CoV-2 genomes are highlighted in purple. Leader regions and the positions of the different viral proteins are also shown along the genome. Source data are provided as a Source Data file.

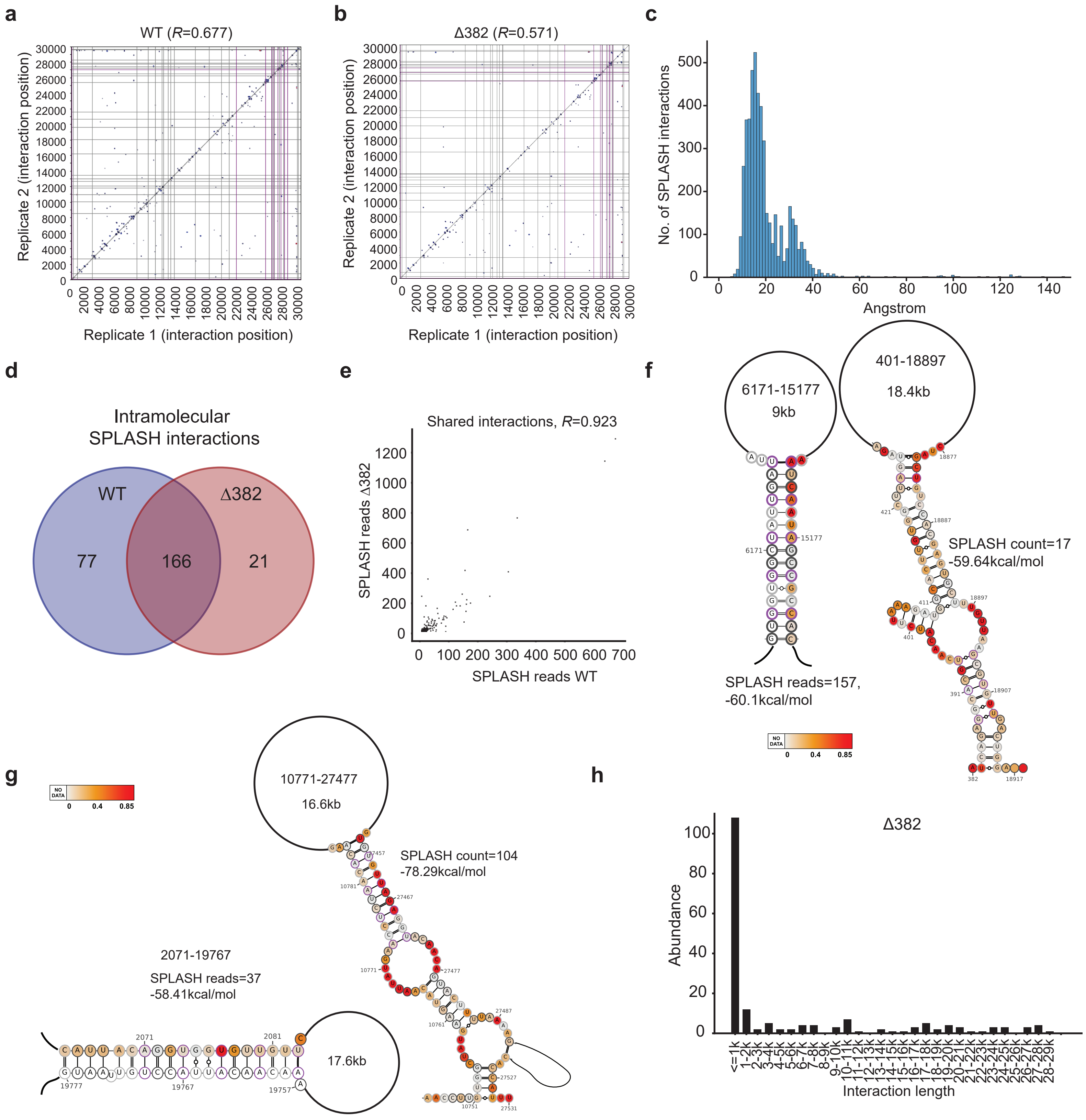

Supp. Figure 7. SPLASH identifies pair-wise RNA interactions within the SARS-CoV-2 genome. a,b 2D matrices showing the location of pair-wise intramolecular RNA interactions between 2 biological replicates of WT (**a**) and Δ382 (**b**) SARS-CoV-2 genomes. **c** Histogram showing the distribution of the spatial distance captured by SPLASH chimeras in 18S and 28S rRNA. 82.9% of our SPLASH chimeric interactions on 18S and 28S rRNA interactions fall within 30A. **d** Venn diagram showing the overlap in intramolecular pair-wise RNA-RNA interactions in WT and Δ382 genome, using SPLASH. **e** Scatterplot showing the correlation in read count for the shared 166 chimeric interactions in WT and Δ382 genomes. **f,g** Structure models of pair-wise RNA interactions are generated using RNAcofold. SHAPE-MaP reactivities are mapped onto the modelled secondary structures. The SPLASH chimeric counts and predicted interaction energy for each model is also shown. **h** Histogram showing the distribution of interactions that span different lengths along the Δ382 SARS-CoV-2 genome. Interactions over distance longer than 1kb are classified as "long-range" and comprise 42.25% of all interactions. Source data are provided as a Source Data file.

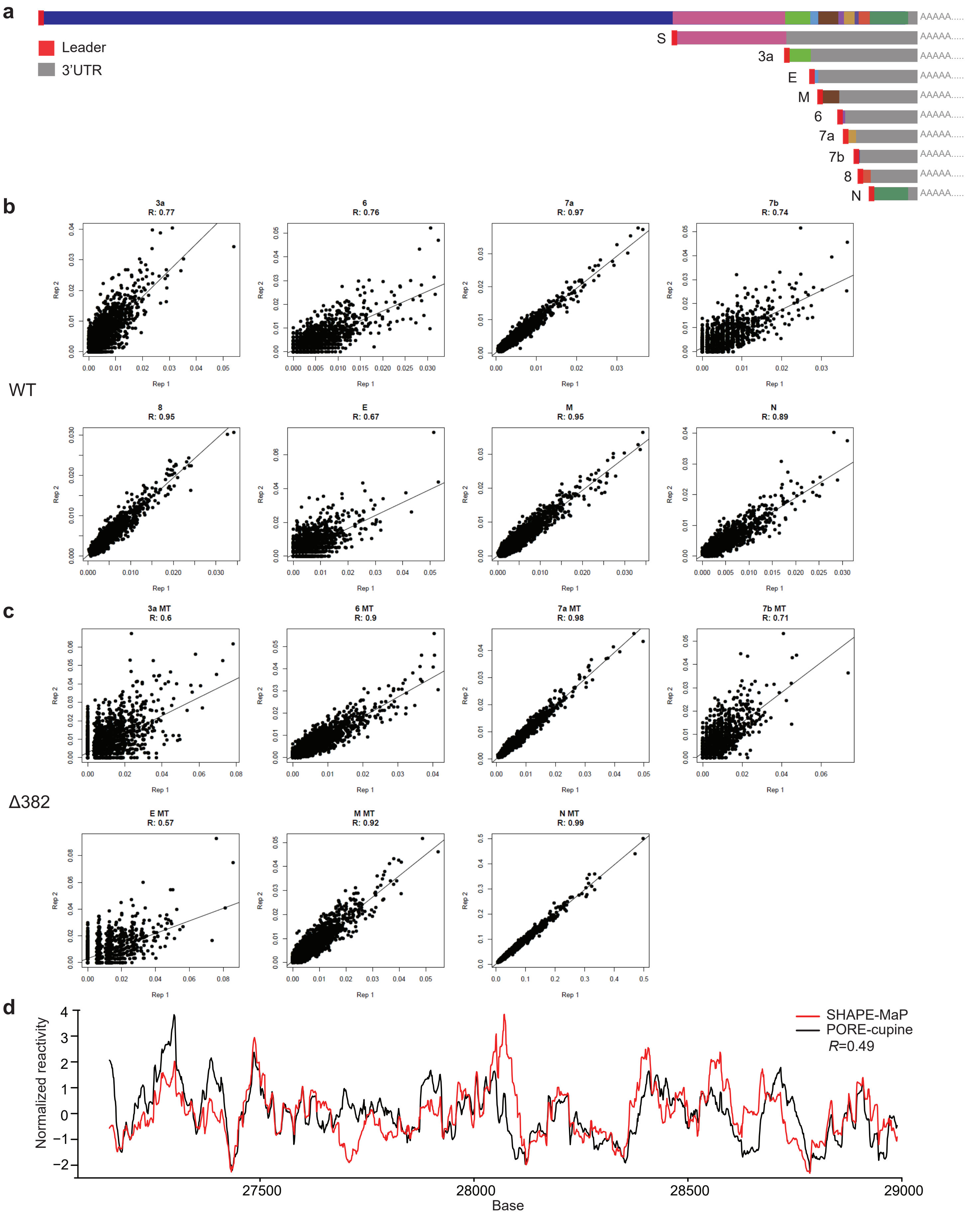

Supp. Figure 8. PORE-cupine reactivities provide information on sgRNAs. a Schematic of the SARS-CoV-2 sgRNAs expressed inside cells. **b,c** Scatter plots of the PORE-cupine reactivities between 2 biological replicates for each sgRNAs from WT (**b**) and Δ382 (**c**) SARS-CoV-2 genomes. **d** Line plots showing the structure reactivity along SARS-CoV-2 obtained using SHAPE-MaP (red) and PORE-cupine (black). *R* (Pearson correlation) between the two plots is 0.49. Source data are provided as a Source Data file.

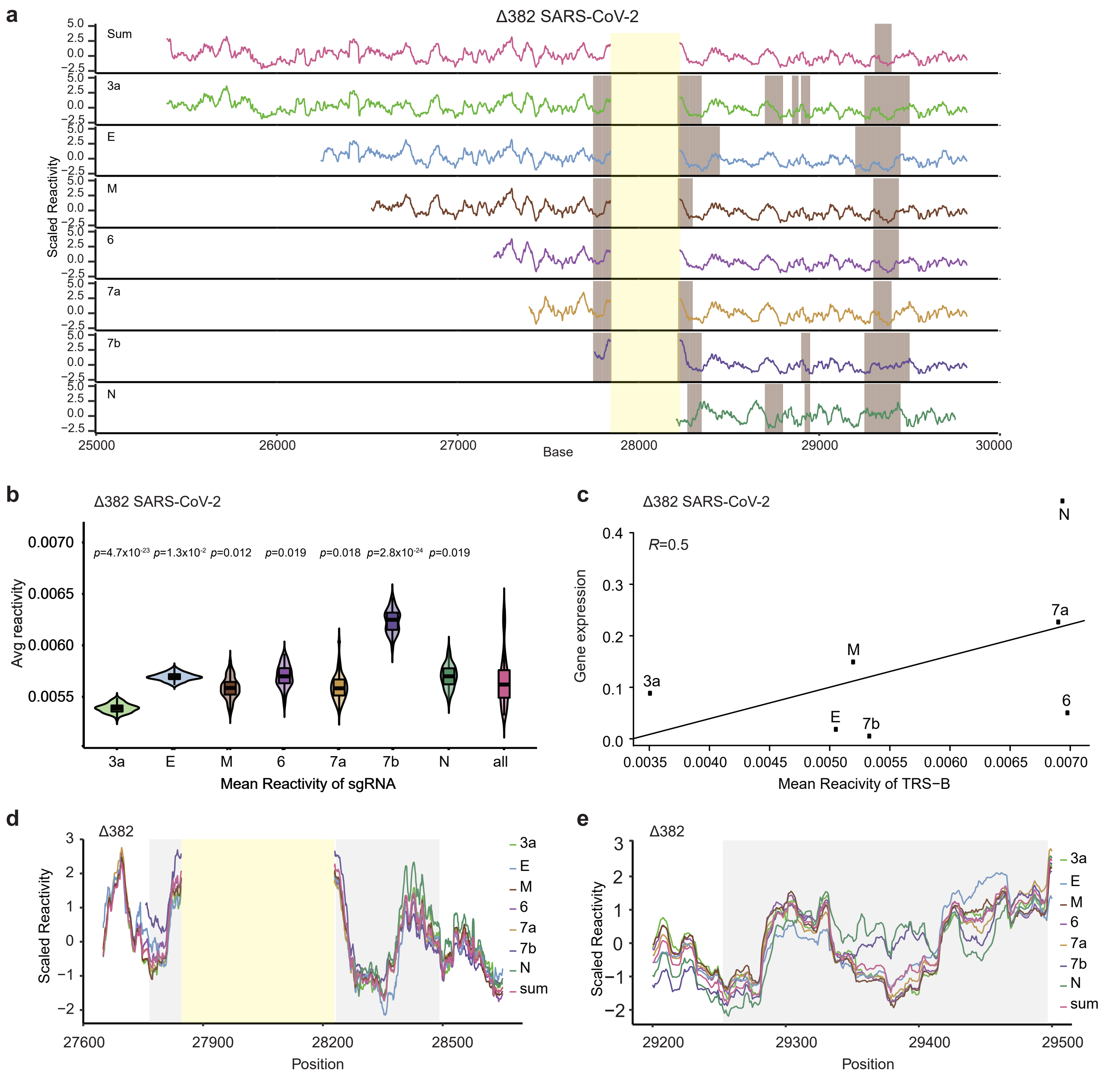

Supp. Figure 9. PORE-cupine reactivities along the sgRNAs of Δ382 SARS-CoV-2. a PORE-cupine reactivity signals are averaged across all of the signals from the sgRNAs (Sum). PORE-cupine reactivity signals are also shown for 3a (dark green), E (blue), M (brown), 6 (purple), 7a (light brown), 7b (navy), 8 (red) and N (dark green). PORE-cupine reactivity signals for each sgRNA are filtered for full length sequences that contain leader sequences for each sgRNA. Regions with significant differences are highlighted in grey ($p<0.05$) (Methods). **b** Violin plots showing the distribution of average reactivities for each sgRNA. Each sgRNA is subsampled for 500 strands before calculating its mean, n = 100. P-values were calculated by comparing the distribution of the reactivities in each sgRNA against all of the sgRNAs with two-sided Wilcoxon Rank Sum test. The box represents the 25–75th percentiles, and the median is indicated. The whiskers show the minimum and maximum values **c** Scatterplot showing the correlation between the PORE-cupine reactivity around TRS-B for each sgRNA (x-axis) against transcript levels inside cells (y-axis). **d,e** Line plots of the PORE-cupine reactivities of the different sgRNAs. **d** P-value for the follow regions are: 27800- 27900 (p-value $=4.92 \times 10^{-7}$), 28250-28350 (p-value = 0.0001), 28300-28400 (p-value = 0.003), 28350-28450 (p-value = 0.0009) and 28400-28500 (p-value = 0.02). **e** P-value for the follow regions are: 29250-29350 (p-value = 0.04), 29300-29400 (p-value = 0.001), 29350-29450 (p-value $= 4.28 \times 10^{-6}$), 29400-29500 (p-value = 0.0001), 29450-29550 (p-value = 0.037). Source data are provided as a Source Data file.

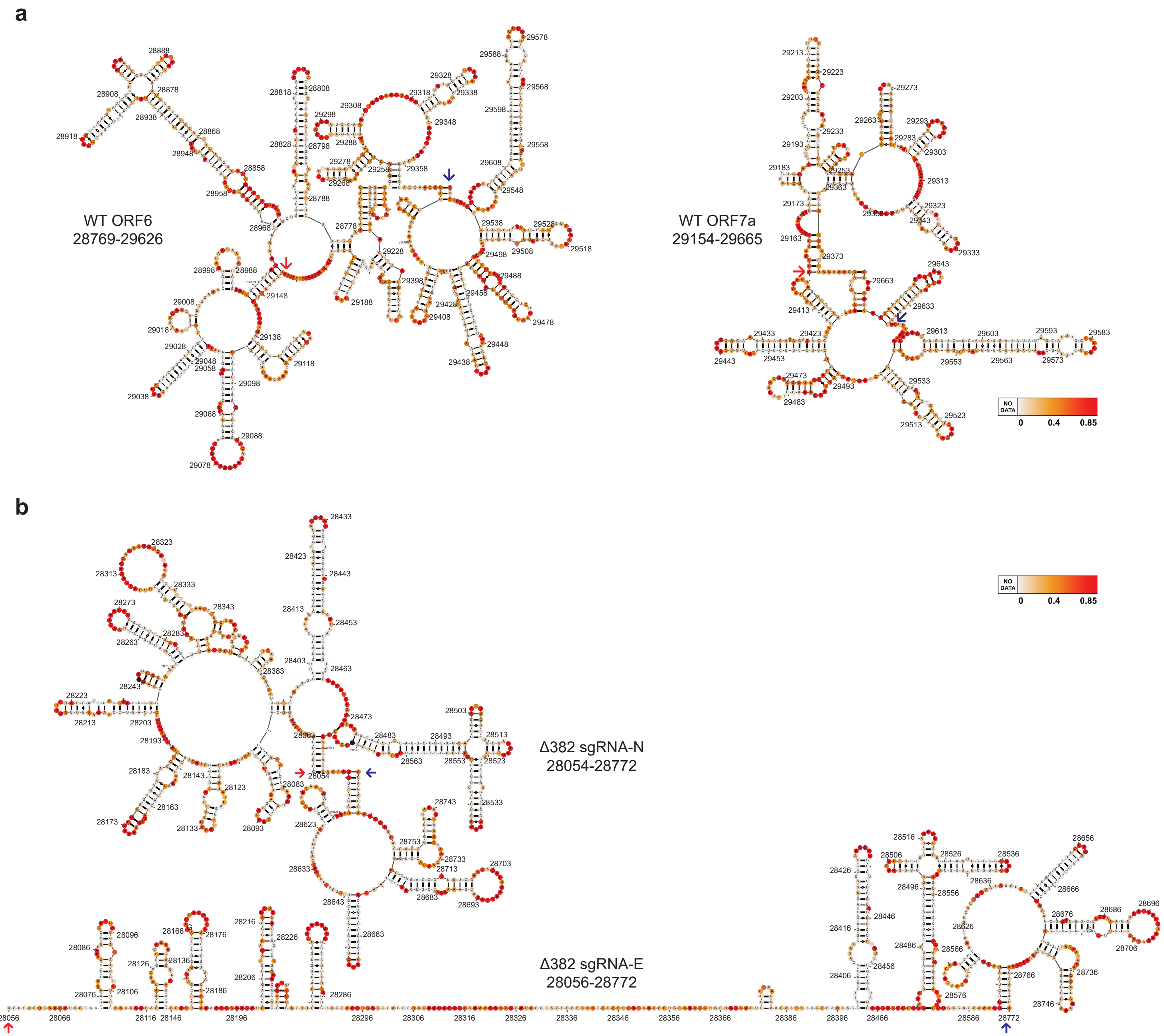

Supp. Figure 10. Structure models of SARS-CoV-2 sgRNAs. a,b Structure models of WT ORF6 and ORF7a (**a**) and Δ382 sgRNA N and E (**b**) are generated using the program RNAstructure, using PORE-cupine reactivities as constraints. PORE-cupine reactivities are mapped onto the secondary structure models. The red and blue arrows indicate the exact same start (red) and stop (blue) positions for the two structure models.

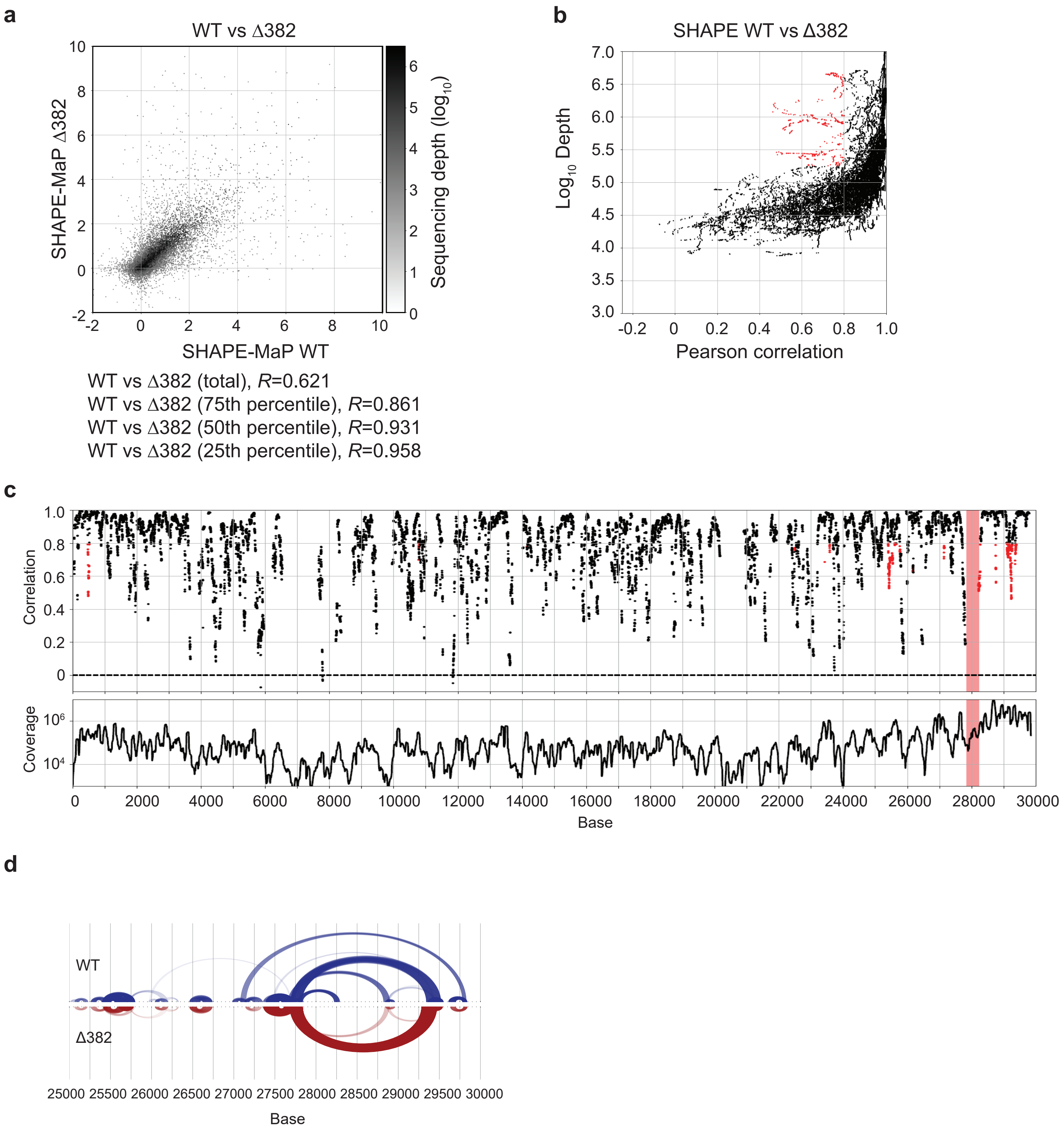

Supp. Figure 11. SHAPE-MaP reactivity differences between WT and Δ382 genomes. **a** Scatterplot of SHAPE-MaP reactivities for WT and Δ382. *R* (Pearson correlation)= 0.621. **b** Scatter plot showing read abundance (Y-axis) and Pearson correlation of SHAPE-MaP reactivity (X-axis). Regions with high read depth but low correlation are highlighted in red. **c** Top, Line plot showing Pearson correlation of SHAPE-MaP reactivities between WT and Δ382 genomes. Bottom, read depth of the sequencing coverage along the genome. Regions that have high read depth and low correlation in terms of reactivities are shown in red. **d** Arc plots showing the pair-wise RNA interactions around the Δ382 deletion site for WT (blue, top) and Δ382 (red, bottom) genomes. Source data are provided as a Source Data file.

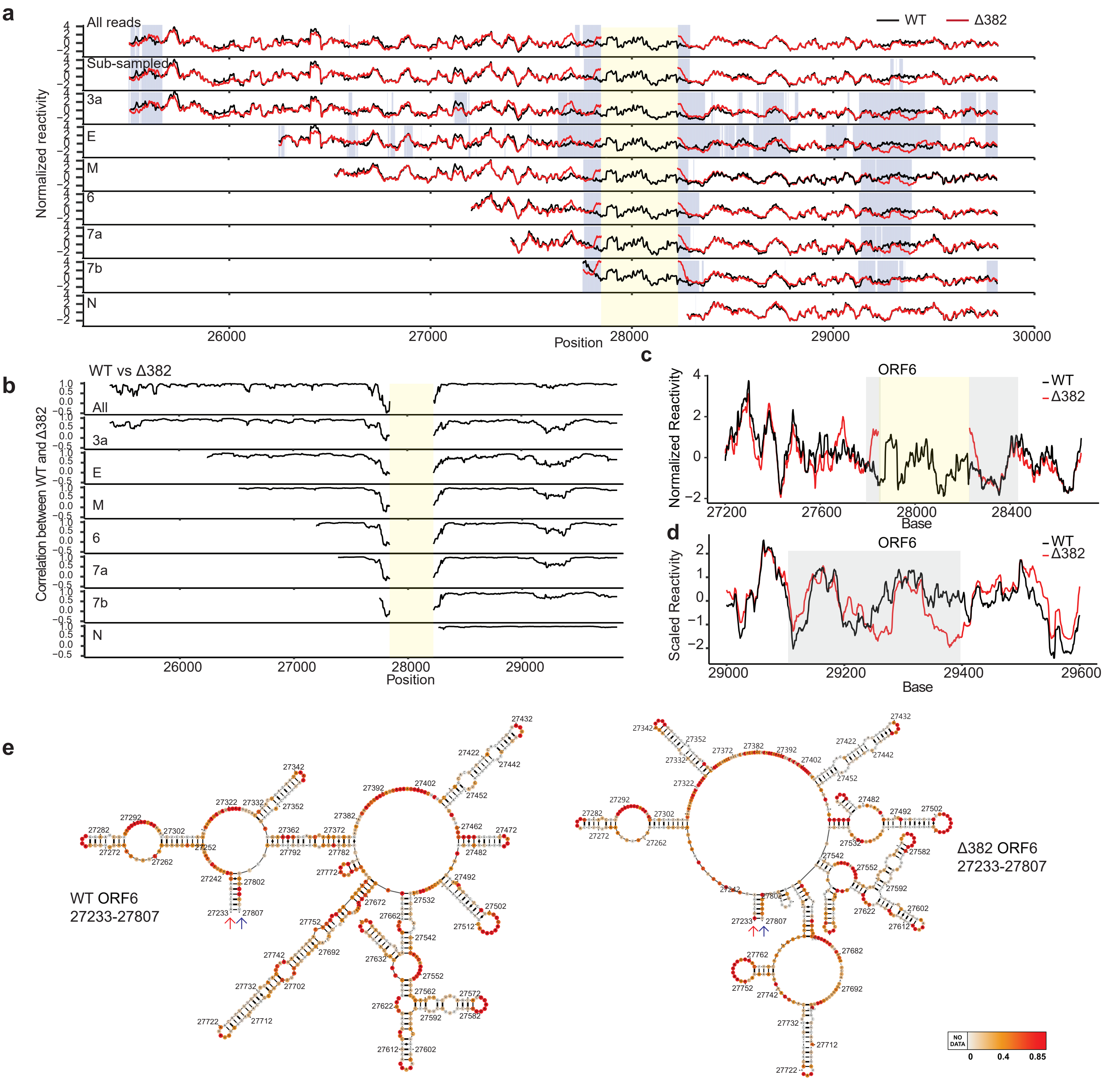

Supp. Figure 12. PORE-cupine reactivities differences between WT and Δ382 SARS-CoV-2 sgRNAs. a PORE-cupine reactivity signals for WT (black) and Δ382 (red) are averaged across all the signals from their respective sgRNAs (All reads).
Line plots representing the sub-sampled lane are the averaged WT (black) and Δ382 (red) signals across the different sgRNA after the sgRNAs have been subsampled to the same depth, and hence carry equal weightage to each other. PORE-cupine reactivity signals for WT (black) and Δ382 (red) are also shown for sgRNAs 3a, E, M, 6, 7a, 7b, and N. PORE-cupine reactivity signals for each sgRNA is filtered for full length sequences that contain leader sequences for each sgRNA. Regions that show significant differences between WT and Δ382 sgRNAs are highlighted in blue, (Methods). **b** Pearson correlation between WT and Δ382 PORE-cupine reactivities for sum of all sgRNAs for WT and Δ382 and between individual sgRNAs of WT and Δ382 genomes. **c,d** Line plots showing the PORE-cupine reactivity of ORF6 along WT and Δ382 around the Δ382 deletion (between 27200-28600, **c**) and 1 kb downstream of the deletion (29000-29600, **d**). **c** *P*-value for the follow regions are: 27750- 27850 (p-value = 0.002), 27800-27900 (p-value = 5.9×10^{-9}), 28250-28350 (p-value = 5.36×10^{-12}), 28300-28400 (p-value = 1.04×10^{-7}) and 28350-28450 (p-value = 2.85×10^{-5}). **d** *P*-value for the follow regions are: 29100-29200 (p-value = 0.02), 29150-29250 (p-value = 0.008), 29200-29300 (p-value = 0.01), 29250-29350 (p-value = 0.04). **e** Structure models of WT and Δ382 ORF6 are generated using the program RNAstructure, using PORE-cupine reactivities as constraints. PORE-cupine reactivities are mapped onto the secondary structure models. Source data are provided as a Source Data file.

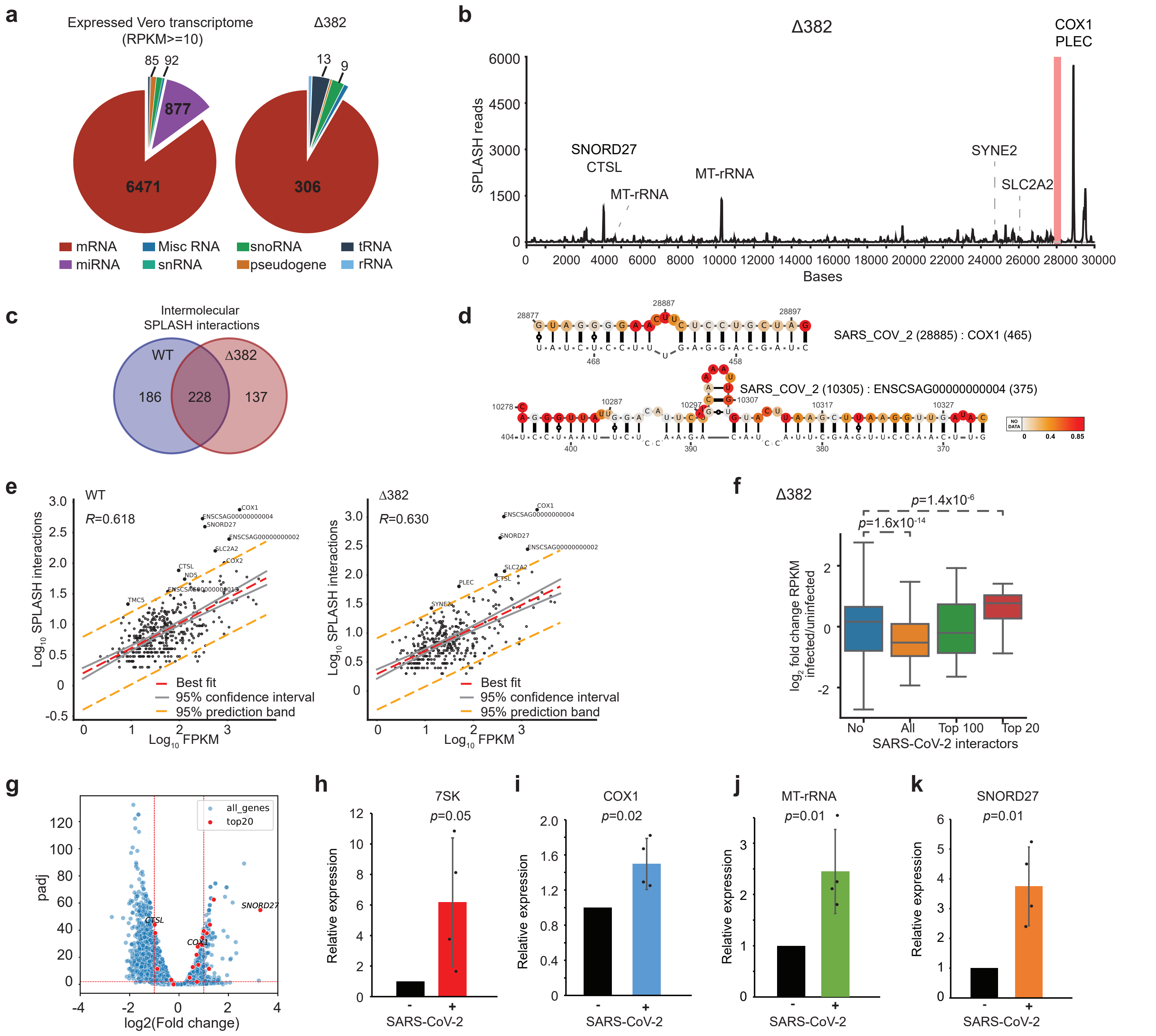

Supp. Figure 13. SARS-CoV-2: host RNA interactions. a Pie-chart showing the distribution of expressed RNAs in Vero-E6 cells (left) and RNAs that interact with Δ382 SARS-CoV-2 (right) in different RNA classes. **b** Line plot showing the number of SPLASH reads along the Δ382 SARS-CoV-2 genome. The names of host RNAs that bind strongly to the virus at a particular location is labelled above the interaction peak. The pink box indicates the deletion region in Δ382. **c** Venn diagram showing the overlap of intermolecular interactions between virus and host in WT and Δ382 genomes. **d** Structure models of pair-wise virus-host RNA interactions are identified using SPLASH and generated using the program RNAcofold. **e** Scatterplot showing the correlation between virus-host SPLASH interactions and host cellular abundance for WT (left) and Δ382 (right) genome. The orange line indicates the best fit line. The grey line indicates the 95% confidence interval of the best fit line. The yellow line indicates 2 standard deviations from the best fit line. Dots above the yellow line have higher SPLASH interactions with virus than would be expected from their abundance. **f** Boxplots showing the distribution of log2 fold change in gene expression upon Δ382 SARS-CoV-2 infection in all non-interacting genes, in (All) RNAs that interact with Δ382 (*n* = 334), in the top 100 Δ382 interactors and top 20 Δ382 interactors. Δ382 SARS-CoV-2 interactors show a decrease in gene expression upon virus infection. However, the top interactors show an increase in gene expression upon virus infection, indicating that they are selectively stabilized. *P*-value was calculated by two-tailed Wilcoxon Rank Sum test. The box represents the 25–75th percentiles, and the median is indicated. The whiskers show the minimum and maximum values. **g** Volcano plot showing the distribution of host RNA gene expression upon Δ382 SARS-CoV-2 infection. The top 20 Δ382 interactors are highlighted in red and show a general stabilization in gene expression upon virus infection. **h-k** qPCR results of host RNAs in Vero-E6 cells in the absence and presence of SARS-CoV-2. Data are presented as mean ± SD (average values from *n* = 2 biological replicates measured and four technical replicates are plotted). *P*-value was calculated using two-tailed *t*-test. Source data are provided as a Source Data file.

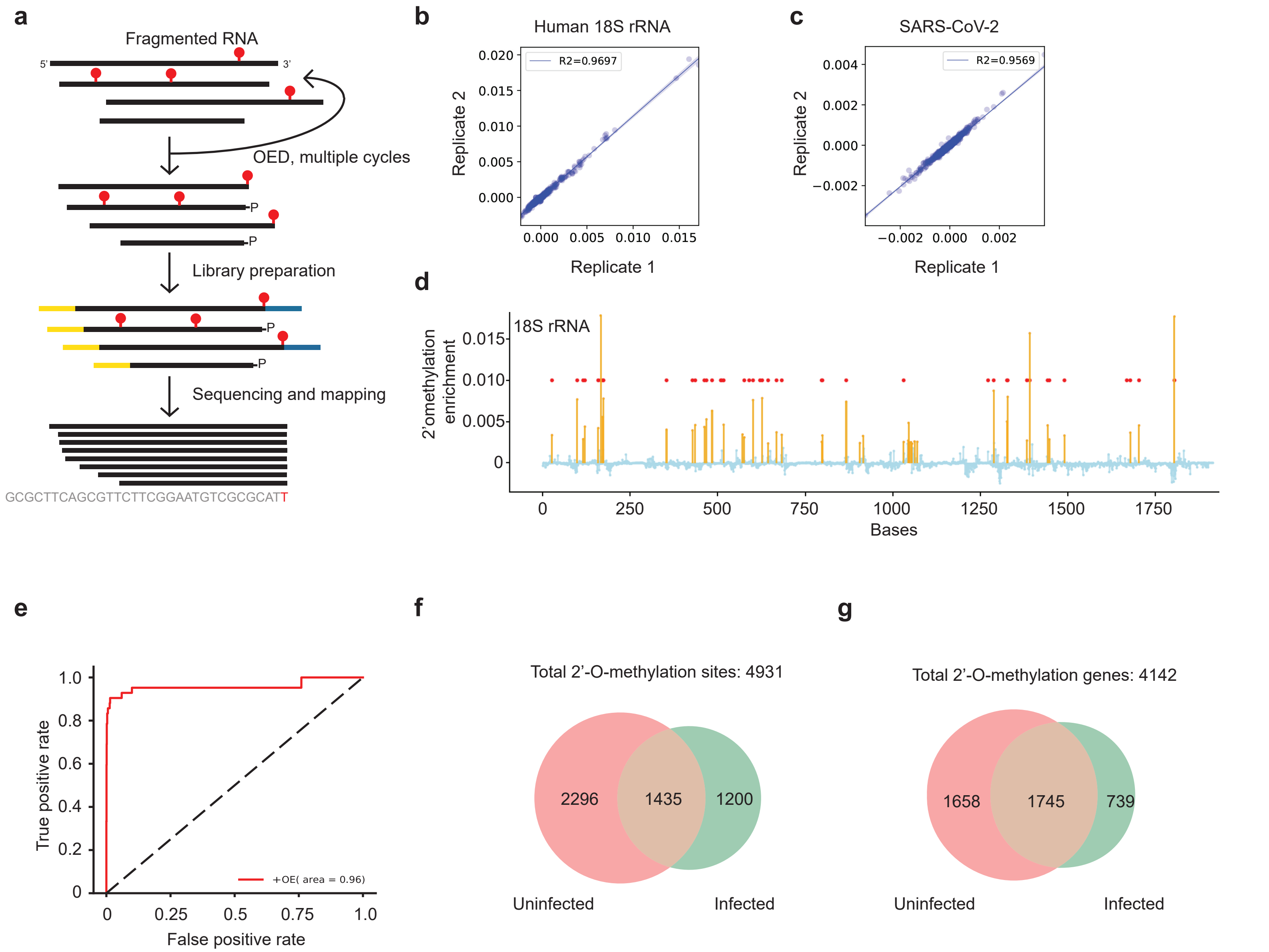

Supp. Figure 14. Nm-seq identifies 2'-O-methylation sites on SARS-CoV-2 and Vero-E6 transcriptome. a Schematic of the Nm-Seq protocol to identify 2'-O-methylation sites in SARS-CoV-2 infected Vero-E6 cells. Red dots represent 2'-O-methylation sites. OED: oxidation-elimination-dephosphorylation. **b** Scatter plot showing the distribution of 2'-O-methylation sites along 18S rRNA in (n = 2) biological replicates of HeLa total RNA. **c** Scatter plot showing the distribution of 2'-O-methylation sites along SARS-CoV-2 in (n = 2) biological replicates of virus infected Vero-E6 cells. **d** The distribution of 36 identified 2'-O-methylation sites along human 18S rRNA. The orange bars indicate enriched sites above control. The red dots indicate known 2'-O-methylation sites. **e** AUC/ROC curve of identified 2'-O-methylation sites on human 18S rRNA. **f,g** Venn diagrams showing the number of sites (**f**) and transcripts (**g**) that are 2'-O-methylated in Vero-E6 transcriptome with and without SARS-CoV-2 infection. Source data are provided as a Source Data file.

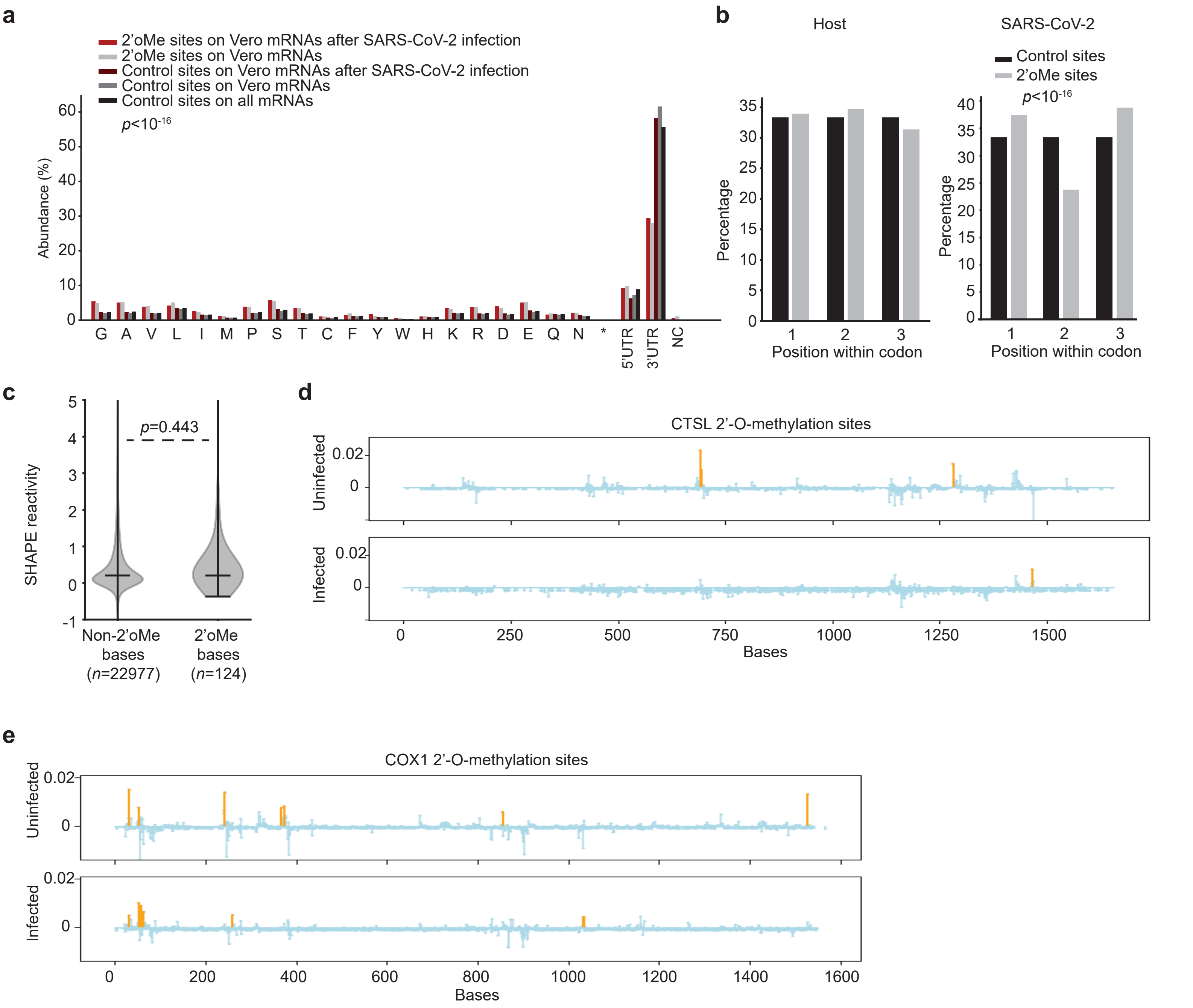

Supp. Figure 15. Distribution of 2'-O-methylation sites on SARS-CoV-2 and Vero-E6 transcriptome. a Bar charts showing the distribution of 2'-O-methylation sites, and control sites, in different amino acids, 5' UTR, 3' UTR and non-coding regions (NC) on the Vero-E6 transcriptome. *P*-value is calculated using chi-squared test. 2'-O-methylation sites are enriched in charged amino acids. **b** Bar charts showing the distribution of 2'-O-methylation sites along positions 1,2 and 3 of codons in the Vero-E6 transcriptome (left) and on SARS-CoV-2 (right). 2'-O-methylation sites are depleted on the 2nd position of codons in SARS-CoV-2. *P*-value was calculated using one-way chi-squared test without adjustments. **c** Violin plot showing the distribution of SHAPE-MaP reactivities on SARS-CoV-2 bases that are not 2'-O-methylated (n = 22977) and are 2'-O-methylated (n = 124). Significance was assessed using two-sided Wilcoxon Rank Sum test without adjustments. **d,e** Distribution of 2'-O-methylation sites on CTSL (**d**) and COX1 (**e**) along its transcript, in uninfected (top) and infected (bottom) Vero-E6 cells. Source data are provided as a Source Data file.

Supplementary Table 1. List of primers

Primer name	Sequence
CS_COX1_F901	ACACGGGCCTACTTTACCTCTG
CS_COX1_R1021	CTCAAAGTATTGCGGCAGATC
CS_7SK_F91_qPCR2	TTCCTGCCTCACCACTTCAT
CS_7SK_R230_qPCR2	TGAGAGCTCGTTTGGAGGTT
CS_SNORD27_F22_qPCR3	GACAAGCATATGGCTGAAC
CS_SNORD27_R71_qPCR3	CTTCTCAGTAGTAACATGAC
CS_ENS004-F1001	ATTGGCCTGTCCGTGAAGAGA
CS_ENS004-R1169	TGTTCGAGTTGACTGTGCTCCG
CS_bACTIN-F136	GGCATGGGTCAGAAGGATTCA
CS_bACTIN-R274	TGTAGAAGGTGTGGTGCCAGAT
