# Supplementary material for: Comprehensive mapping of SARS-CoV-2 interactions in vivo reveals functional virus-host interactions
Source: Nat Commun. 2021 Aug 25;12:5113. doi: 10.1038/s41467-021-25357-1 (PMC8387478; doi:10.1038/s41467-021-25357-1)
Supplement: Supplementary file 3 — Description of Additional Supplementary Information [file 41467_2021_25357_MOESM3_ESM.pdf]

Description of Additional Supplementary Files

File Name: Supplementary Data 1

Description: Statistics for next-generation sequencing of SHAPEMaP, PORE-cupine, SPLASH and Nm-Seq.

File Name: Supplementary Data 2

Description: SHAPE-MaP data for WT and Δ382 SARS-CoV-2.

File Name: Supplementary Data 3

Description: Consensus regions with high SHAPE-MaP reactivities (top 20%) in WT and Δ382 SARS-CoV-2.

File Name: Supplementary Data 4

Description: SPLASH pair-wise intramolecular interactions along WT SARS-CoV-2 genome.

File Name: Supplementary Data 5

Description: SPLASH pair-wise intramolecular interactions along Δ382 SARS-CoV-2 genome.

File Name: Supplementary Data 6

Description: Publicly available SARS-CoV-2 ribosome profiling data that is used in this study.

File Name: Supplementary Data 7

Description: PORE-cupine reactivities in WT and Δ382 SARS-CoV2 genomes.

File Name: Supplementary Data 8

Description: SPLASH interactions between host cell RNAs and Δ382 SARS-CoV-2.

File Name: Supplementary Data 9

Description: SPLASH interactions between host cell RNAs and WT SARS-CoV-2.

File Name: Supplementary Data 10

Description: 2’-O-methylation sites in Vero-E6 RNAs with and without SARS-CoV-2 infection.

File Name: Supplementary Data 11

Description: 2’-O-methylation sites in SARS-CoV-2 genome.
